# Mercury Exposure in Ireland: Results of the DEMOCOPHES Human Biomonitoring Study

**DOI:** 10.3390/ijerph110909760

**Published:** 2014-09-17

**Authors:** Elizabeth Cullen, David S. Evans, Fred Davidson, Padraig Burke, Damien Burns, Andrew Flanagan, Chris Griffin, Anne Kellegher, Rory Mannion, Maurice Mulcahy, Michael Ryan, Pierre Biot, Ludwine Casteleyn, Argelia Castaño, Jürgen Angerer, Holger M. Koch, Marta Esteban, Birgit K. Schindler, Carmen Navarro, Marike Kolossa-Gehring, Ulrike Fiddicke, Greet Schoeters, Elly Den Hond, Ovnair Sepai, Karen Exley, Louis Bloemen, Lisbeth E. Knudsen, Reinhard Joas, Anke Joas, Dominique Aerts

**Affiliations:** 1 Department of Community of Health, Health Service Executive, Kildare, Ireland; 2 Department of Public Health, Health Service Executive, Galway, Ireland; E-Mail: david.evans@hse.ie; 3 Public Analyst’s Laboratory Health Service Executive, Cork, Ireland; E-Mail: fred.davidson@hse.ie; 4 Public Analyst’s Laboratory, Health Service Executive, Galway, Ireland; E-Mails: padraig.burke@hse.ie (P.B.); andrew.flanagan@hse.ie (A.F.); rory.mannion@hse.ie (R.M.); 5 Project Manager, Health Service Executive, Palmerstown, Dublin 20, Ireland; E-Mail: damien.burns@hse.ie; 6 Public Analyst’s Laboratory, Health Service Executive, Dublin 2, Ireland; E-Mail: chris.griffin@hse.ie; 7 Environmental Health Service, Health Service Executive, Leitrim, Ireland; E-Mail: ann.kellegher@hse.ie; 8 Environmental Health Service, Health Service Executive, Galway, Ireland; E-Mail: maurice.mulcahy@hse.ie; 9 Conway Institute of Biomolecular and Biomedical Research, University College Dublin 4, Ireland; E-Mail: michael.p.ryan@ucd.ie; 10 Federal Public Service Health, Food Chain Safety and Environment, Brussels 1060, Belgium; E-Mails: pierre.biot@environnement.belgique.be (P.B.); dominique.aerts@milieu.belgie.be (D.A.); 11 University of Leuven, Leuven 3000, Belgium; E-Mail: ludwine.casteleyn@med.kuleuven.be; 12 Instituto de Salud Carlos III, Environmental Toxicology Centro Nacional de Sanidad Ambiental (CNSA), Majadahonda, Madrid 28220, Spain; E-Mails: castano@isciii.es (A.C.); m.esteban@isciii.es (M.E.); carnavarro@isciii.es (C.N.); 13 Institute for Prevention and Occupational Medicine of the German Social Accident Insurance, Institute of the Ruhr-Universität Bochum (IPA), Bochum 44789, Germany; E-Mails: angerer@ipa-dguv.de (J.A.); koch@ipa-dguv.de (H.M.K.); Birgit.schindler@proof-acs.de (B.S.); 14 Federal Environment Agency, Berlin 14195, Germany; E-Mails: marike.kolossa@uba.de (M.K.-G.); Ulrike.Fiddicke@uba.de (U.F.); 15 Flemish Institute of Technological Research, Environmental Risk and Health Unit, MolB-2400, Belgium; E-Mails: greet.schoeters@vito.be (G.S.); elly.denhond@vito.be (E.D.H.); 16 Centre for Radiation, Chemical and Environmental Hazards, Public Health England, Chilton OX11 ORQ, Oxfordshire UK; E-Mails: ovnair.sepai@phe.gov.uk (O.S.); Karen.Exley@phe.gov.uk (K.E.); 17 Environmental Health Science International, 4561 HV Hulst, The Netherlands; E-Mail: lb@ehsi.eu; 18 21 Department of Public Health, University of Copenhagen, Copenhagen 1353, Denmark; E-Mail: liek@sund.ku.dk; 19 BiPRO GmbH, Munich 81545, Germany; E-Mails: reinhard.joas@bipro.de (R.J.); anke.joas@bipro.de (A.J.)

**Keywords:** mercury, human biomonitoring, hair, exposure

## Abstract

*Background*: Monitoring of human exposure to mercury is important due to its adverse health effects. This study aimed to determine the extent of mercury exposure among mothers and their children in Ireland, and to identify factors associated with elevated levels. It formed part of the Demonstration of a study to Coordinate and Perform Human Biomonitoring on a European Scale (DEMOCOPHES) pilot biomonitoring study. *Methods*: Hair mercury concentrations were determined from a convenience sample of 120 mother/child pairs. Mothers also completed a questionnaire. Rigorous quality assurance within DEMOCOPHES guaranteed the accuracy and international comparability of results. *Results*: Mercury was detected in 79.2% of the samples from mothers, and 62.5% of children’s samples. Arithmetic mean levels in mothers (0.262 µg/g hair) and children (0.149 µg /g hair) did not exceed the US EPA guidance value. Levels were significantly higher for those with higher education, and those who consumed more fish. *Conclusions*: The study demonstrates the benefit of human biomonitoring for assessing and comparing internal exposure levels, both on a population and an individual basis. It enables the potential harmful impact of mercury to be minimised in those highly exposed, and can therefore significantly contribute to population health.

## 1. Introduction

Mercury is one of the most toxic metals in the environment and represents a significant threat to human and environmental health [1]. It is found in air, water, and soil, occurring both naturally and as a result of emissions from human activities. Mercury exposure may also result from products containing mercury (e.g., thermometers, batteries, fluorescent lights) or accidentally from a chemical incident [2]. Mercury exposure for the general population arises largely from fish and shellfish consumption [3]. The most toxic form of mercury is methyl mercury, found in significant levels in fish and seafood [4]. This is formed when mercury (which ultimately settles in the aquatic environment) is converted by micro-organisms into methyl mercury. It is absorbed by phytoplankton, and its concentration in organisms rises as the marine food chain is ascended, accumulating in long lived predatory fish such as swordfish, tuna and shark [3]. Cod, whiting and pike are also contributors to the adult diet, with hake also contributing to children's diets [5].

Mercury is generally excreted at a slower rate than it is ingested, and therefore can bio-accumulate in the body. Excessive exposure can cause neurological problems, with symptoms including tremors, memory loss, neuromuscular effects, headaches, cognitive and motor dysfunction [3]. The digestive and immune systems, lungs, kidneys, skin and eyes may also be affected [3]. Children are the most vulnerable [6]. As methyl mercury passes both through the placenta and the blood-brain barrier [7], the developing brain is believed to be the most sensitive [4]. Cognitive thinking, memory, attention, language, fine motor and visual spatial skills can be affected in children exposed to methyl mercury as foetuses [3,8,9]. Mental retardation, seizures, vision and hearing loss, delayed development, language disorders and memory loss may also occur. There is also evidence of reduced birth weight with prenatal exposure to mercury [8].

Due to the adverse health effects of mercury it is important that exposure levels are monitored to assess and develop strategies to reduce risk. Globally, a number of studies have assessed human exposure to mercury in countries such as the USA [10], Canada [11], China [12], and in countries with high fish consumption such as the Faroe Islands [13], the Seychelles [14], and in the Arctic region [9,15,16]. Although there have been studies undertaken in Europe [17,18] there is a relative absence of standardized comparable data for the region [19]. In Ireland, mercury levels in the food chain are regularly assessed. However, as mercury exposure may result from a wide variety of different sources, it is difficult to estimate overall human exposure levels. This issue can be overcome with human biomonitoring, which involves collecting samples from humans (e.g., blood, urine, hair, saliva), to assess chemical contamination from all sources of exposure. At the time of the study, Ireland did not have a national human biomonitoring programme. The present study was developed as a result of the European Commission’s recognition of the need to develop a coherent approach to biomonitoring in Europe [20]. It formed part of the Demonstration of a study to Coordinate and Perform Human Biomonitoring on a European Scale (DEMOCOPHES) pilot study and involved the biomonitoring of four key environmental pollutants (mercury, cadmium, cotinine and phthalates) in 17 countries throughout Europe [21]. The aim of this study was to determine the extent of mercury exposure among mothers and their children in Ireland and to identify factors associated with elevated levels.

## 2. Experimental Section

A convenience sample of two locations was selected (urban and rural). Twelve schools in these locations (eight urban and four rural) were asked to participate in the study. Selection was based on the need to include participants from all socioeconomic groups. In addition, preference was given to larger schools to ensure a sufficient number of responses. Eight schools (four urban and four rural) subsequently agreed to participate in the study. An information pack containing an invitation letter and reply card (for parents) was distributed to all children aged between 6 and 11 years attending these schools to request participation. To be eligible to participate, mothers had to be 45 years of age or less and to have resided in the study area for at least five years. Positive replies were randomly contacted by telephone to confirm eligibility and organise sample collection until a quota of 120 mother/child pairs was achieved (60 urban and 60 rural). These participants were visited by Environmental Health Officers in their homes (October 2011–January 2012) to collect hair samples from mothers and children. Mothers also completed an interviewer led questionnaire to obtain socio-demographic information, exposure to mercury, and information on diet. Following data collection, a gift voucher was presented to the mother as a gratuity. Hair samples were analysed by the Public Analyst Laboratory (Health Service Executive, Cork), employing a validated and accredited operating procedure. All samples were analysed using an AMA-254 Automated Mercury Analyser. Certified Reference Materials (CRMs) were used in the analyses for quality control purposes. Quality standards were assured at the laboratory by the successful completion of the DEMOCOPHES quality assurance programme [22] (a requirement of the DEMOCOPHES project). The limit of mercury detection (LoD) was 0.022 µg/g, and the limit of quantification (LoQ) was 0.07 µg/g. Values below the detection limit were replaced with half the LoQ value. As the data for hair mercury were not normally distributed, non parametric tests including Spearman’s rho, Chi square, Kruskal-Wallis H tests, and Mann-Whitney U tests were undertaken. In addition, standard linear multiple regression analysis (using the stepwise method) was conducted to assess the key predictors associated with mercury exposure. For the multiple regression analysis mercury levels were log transformed to normalize the data. Data analysis was undertaken using IBM SPSS Statistics V20 (Presidion [formerly SPSS Ireland], Dublin, Ireland). All subjects gave their informed consent for inclusion before they participated in the study. Ethical approval for the protocol was obtained from the Research Ethics Committee, Faculty of Public Health Medicine (Royal College of Physicians of Ireland, 23 September 2011). The study was conducted in accordance with the Declaration of Helsinki, the Oviedo Convention and its Additional Protocol concerning biomedical research, and with the European Data Protection Directive 95/46/EC.

## 3. Results and Discussion

### 3.1. Response Rate

Of the 1835 invitation letters (1185 urban and 650 rural), replies were received from 551 families (30% response rate; 20% urban and 50% rural). Of these, 311 were positive (142 urban and 169 rural; 12% and 26% of invitation letters, respectively). A further 33 families were excluded during quota sampling as they did not meet the eligibility criteria. Following quota sampling, 120 mother / child pairs (60 urban and 60 rural) were subsequently included in the study (6.5% of the families contacted).

### 3.2. Profile

Details of the study population are outlined in Table 1. Almost two thirds of mothers (63%) were aged 40 years or younger (mean = 38.1, median = 39.0). A total of 13% were single, and over half (53%) had received tertiary education. The majority of mothers (59%) and fathers (79%) worked outside the home. Over a quarter (29%) of mothers smoked, with 43% of households having at least one smoker. A larger proportion of children were boys (53%) with 51% aged 5–8 years (mean = 8.5, median = 8.0).

**Table 1:** Profile of study population.

Profile	No.	%
Area of residence		
Urban	60	50.0
Rural	60	50.0
Highest education level in family		
Primary or lower secondary	8	6.7
Higher secondary or post secondary non tertiary	49	40.8
Tertiary education	63	52.5
Single mothers	16	13.3
Age of mother (years)		
≤35	34	28.3
35.1–40	41	34.2
>40	45	37.5
Age of child (years)		
5–8	61	50.8
9–11	59	49.2
Gender of child		
Boy	63	52.5
Girl	57	47.5
Mothers who smoke	35	29.2
Households with at least one smoker	51	42.5
Working outside home		
Mothers	71	59.2
Fathers	82	78.8

### 3.3. Overall Mercury Exposure

Mercury was detected in 79.2% of mother’s samples and 62.5% of children’s samples (Table 2). This difference is statistically significant (χ^2^ = 19.971, df = 1, *p* < 0.001). In comparing samples from mothers and children, it was found that mercury levels in children were approximately half that of mothers (arithmetic mean = 0.149 µg/g and 0.262 µg/g respectively). These differences were statistically significant (Mann-Whitney U test = 5054.00, *p* < 0.001). The results from three mothers (1.052 µg/g, 1.338 µg/g, 1.460 µg/g) were above the US EPA recommended guidance value for hair (1.0 µg/g) [23]. It is worth noting that all these three mothers had amalgam fillings and two consumed fish several times a week. In addition, one had broken an energy saving lamp at home. Whether these factors contributed to their hair mercury cannot be determined. All other values were below the US EPA [23] guidance value. 

**Table 2:** Analysis of hair mercury in mothers and children.

Key Statistics (µg/g)	Mothers (n = 120)	Children (n = 120)
Percent above limit of quantification (LOQ)	79.20	62.50
Geometric mean	0.165	0.097
Confidence interval	0.137–0.198	0.082–0.114
Arithmetic mean	0.262	0.149
Standard deviation	0.264	0.152
Minimum	0.035	0.035
Maximum	1.460	0.875
10th percentile	0.035	0.035
25th percentile	0.089	0.035
50th percentile	0.188	0.100
90th percentile	0.616	0.352
95th percentile	0.798	0.463
US EPA guidance value [23]	2.0 1.0	

In comparing mercury results by sociodemographic factors, levels significantly increased as a mothers’ education level increased, both for mothers (Kruskal-Wallis H test, χ^2^ = 20.169, df = 2, *p* < 0.001) and children (Kruskal-Wallis H test, χ^2^ = 15.861, df = 2, *p* < 0.001). A positive correlation was found between the mercury levels of mothers and children (Spearman’s rho = 0.615, *p* < 0.001). Mothers less than 35 years of age had significantly lower levels than those over 35 years (Kruskal-Wallis H test, χ^2^ = 9.860, df = 2, *p* < 0.01). In addition, levels were significantly lower in mothers that smoked daily (Kruskal-Wallis H test, χ^2^ = 14.907, df = 3, *p* < 0.01). No significant differences (*p* > 0.05) were found between other sociodemographic variables (e.g., area of residence, working outside the home).

### 3.4. Mercury Levels and Fish and Seafood Consumption

Fish was consumed several times per week by 23% of mothers and 16% of children. Table 3 shows mean hair mercury levels for these mothers and children. All concentrations were below the US EPA (1.0 µg/g) [23] guidance value. The majority of mothers (77%) and children (84%) consumed fish once a week or less often. Higher levels of fish consumption were found in mothers with higher education levels, non smokers, and older mothers. These were only statistically significant for non smokers (χ^2^ = 3.915, df = 1, *p* < 0.05). Mercury levels were significantly higher for mothers (Mann-Whitney U test = 500.50, *p* < 0.001) and children (Mann-Whitney U test = 366.00, *p* < 0.001) who consumed fish several times a week compared to those who consumed fish once a week or less. In terms of the type of fish, significantly higher levels were found for mothers (Mann-Whitney U test = 383.00, *p* < 0.01) and children (Mann-Whitney U test = 307.00, *p* < 0.001) who consumed marine fish several times a week. No significant differences were found in mean mercury levels for mother or children that consumed seafood products (*p* > 0.05). The significance of differences in the consumption of shellfish and freshwater fish could not be computed due to low consumption of these foods.

**Table 3:** Mean hair mercury levels (µg/g) for those who consume fish and seafood several times per week (mothers and children).

	Mothers				Children			
Fish Consumed Several Times per Week	Those above LoQ		Geometric	Arithmetic	Those above LoQ		Geometric	Arithmetic
	No.	%	mean	mean	No.	%	mean	mean
Fish (all types)	28	23.3	0.365	0.442	19	15.8	0.223	0.265
Marine fish	13	10.9	0.314	0.353	13	10.8	0.208	0.232
Shellfish	3	2.8	0.314	0.718	1	0.9	NC ^*^	0.316
Freshwater fish	1	1.0	NC^*^	0.167	3	0.9	NC ^*^	0.232
Other products containing marine fish and shellfish ^**^	6	5.9	0.172	0.188	3	2.8	NC ^*^	0.232

^*^ NC = not computed due to small sample size; ^**^ Products containing marine fish and shellfish (crustacean like shrimps and shell prawns) such as fish or shellfish in soups, fish fingers or sushi, tuna in a salad or on a sandwich/pizza, prawn cocktail, seaweed, *etc.*

### 3.5. Mercury Levels and Other Potential Sources of Exposure

From Table 4, it can be seen that the other potential sources to which the greatest proportion of mothers was exposed include use of a public water supply (91%), having hair toned/dyed in the last six months (83%), and having amalgam teeth fillings (79%).

The potential sources to which the greatest proportion of children were exposed include use of a public water supply (91%), energy saving lamp broken in home (17%) and use of anti lice shampoo in the last six months (16%). All mean mercury levels were below the US EPA (1.0 µg/g) [23] guidance values. The highest mercury levels in mothers were for those who had chemical hair structure treatment in the last six months (n = 5, geometric mean = 0.248, arithmetic mean = 0.310) and those with amalgam teeth fillings (n = 95, geometric mean = 0.178, arithmetic mean = 0.278). For children, the highest mercury levels were for those with amalgam teeth fillings (n = 10, geometric mean = 0.102, arithmetic mean = 0.133) and those who had a mercury thermometer broken at home (n = 6, geometric mean = 0.090, arithmetic mean = 0.110). The sources of exposure with the highest mercury levels were not explained by patterns of fish consumption (*p* > 0.05). For all potential sources of exposure, there were no significant differences in mean mercury levels compared to mothers and children who had not been exposed to these sources (*p* > 0.05).

**Table 4:** Mean hair mercury levels (µg/g) for other sources of exposure (mothers and children)

	Mothers				Children			
Other sources of Exposure	Those above LoQ		Geometric	Arithmetic	Those above LoQ		Geometric	Arithmetic
	No.	%	mean	mean	No.	%	mean	mean
Skin bleaching	2	1.7	0.225	0.237				
Amalgam teeth fillings	95	79.2	0.178	0.278	10	8.3	0.102	0.133
Hair toned/dyed in the last six months	100	83.3	0.159	0.255	4	3.3	0.059	0.067
Chemical hair structure treatment in the last six months	5	4.2	0.248	0.310	1	0.8	NC ^*^	0.035
Anti lice shampoo used in the last six months	14	11.7	0.184	0.213	19	15.8	0.082	0.108
Heavy metals industry in neighbourhood of residence	6	5.0	0.138	0.177	6	5.0	0.083	0.097
Use of well/private water supply	11	9.2	0.174	0.225	11	9.2	0.059	0.080
Use of public water supply	109	90.8	0.164	0.266	109	90.8	0.102	0.156
Mercury thermometer broken in home	6	5.0	0.140	0.150	6	5.0	0.090	0.110
Energy saving lamp broken in home	20	16.7	0.146	0.232	20	16.7	0.075	0.106
Use of soldering iron indoors	7	5.8	0.126	0.210	7	5.8	0.069	0.097

^*^ NC = not computed due to small sample size.

### 3.6. Predictors of Mercury Exposure

For mothers, stepwise multiple regression identified two predictors associated with increased mercury exposure (accounting for 29.7% of the variance). Mothers who consumed all types of fish several times a week or more (standardized beta = 0.387, t = 4.725, *p* < 0.001) and mothers from families with higher education levels (standardized beta = 0.328, t = 3.965, *p* < 0.001) were more likely to have higher exposure levels. These two predictor factors were also identified for children (accounting for 24.1% of the variance). Children that consumed all types of fish several times a week (standardized beta = 0.354, t = 4.418, *p* < 0.001) and children from families with higher education levels were more likely to have higher exposure levels.

### 3.7. Discussion

The study is the first human biomonitoring programme in Ireland to measure exposure to mercury. It forms part of a systematic approach to human biomonitoring across 17 European countries. The testing of hair samples demonstrates that both Irish mothers and children have been exposed to quantifiable amounts of mercury. Mercury is a highly toxic element and ideally adults and children should not have mercury in their bodies [24]. The US EPA [23] have used 1.0 µg/g hair mercury as a guidance value. The mean levels obtained in this study were significantly below this level. In addition, the mean levels of hair mercury detected in both mothers and children were lower than the mean exposure values for Europe recorded in the DEMOCOPHES 17 country study [21], and also lower than those recorded in the US National Health and Nutrition Examination Survey (NHANES) which employed a similar methodology (using hair samples) to assess methyl mercury levels in mothers and children in 1999/2000 [10]. Compared to European studies, levels in the current study are similar for that recorded in Germany [25] and lower that that found in Belgium [26], and the Czech Republic [27].

Although no previous human biomonitoring of mercury has been undertaken in Ireland, food and water is monitored for mercury contamination by the Environmental Protection Agency [22], and the Food Safety Authority of Ireland (FSAI). Whilst it is not possible to assess changes in exposure levels over time, comparisons with levels in food and water do provide evidence to support our findings. A report by the FSAI concluded that mercury levels were generally low, presenting minimal health risk to Irish consumers [6]. A report on drinking water found that mercury was not detected in 99% of samples [28]. Similarly, a report on water quality concluded that mercury was not detected in the vast majority of samples examined [29]. Variations in the biological measures of exposure were observed, with higher hair mercury levels found in mothers compared to children. There was also a positive correlation between the hair mercury levels of mothers and children. Similar patterns have been found in other studies [30,31] with the correlation between mothers and children attributed to similar diets [30]. In addition, higher levels were found in older mothers, those with higher education levels, and non smokers. The US NHANES study found similar exposure patterns by age, although education level differences were not found [10]. Smoking has been found to play a minor role in mercury exposure [32]. Variations in fish consumption may help explain the observed sociodemographic variations and is supported by the multivariate analysis. However, whilst there were differences in fish consumption by sociodemographic factors, these were only statistically significant at the univariate level for non-smoking mothers (who consumed significantly more fish). In addition, the multiple regression analysis revealed that fish consumption and a family’s education level only explained 30% of the variance for mothers and 24% of the variance for children, suggesting that other factors not considered by the study may also be important in explaining mercury exposure levels. These issues warrant a more detailed investigation on a larger sample size before firm conclusions can be drawn.

In terms of fish and seafood consumption, frequent consumers generally had exposure levels below the US EPA [23] guidance value. Tests on fish from Irish ports have also detected low mercury levels (arithmetic mean = 0.08 mg/kg) [33]. Exposure levels for frequent consumers however were significantly higher than those that infrequently consumed fish or seafood. This is consistent with other studies [10] and is explained by the fact that seafood and fish are known to contain significant levels of mercury [3]. Whilst levels were below guidance values [21,23], they do demonstrate the need to monitor exposure from high risk sources. Existing systems of food monitoring by the FSAI, the Public Analyst Laboratories, the Department of Agriculture, Fisheries and Food, and the Marine Institute have been successful at detecting and removing fish products with high mercury levels. For example, a consignment of swordfish was removed from the market in 2012 [34]. This highlights the benefit of human biomonitoring to benchmark mercury exposure. It also reaffirms that fish is a safe product to consume in Ireland, and could be utilised to promote fish consumption which is currently lower than the European average [35,36]. However, as awareness of mercury contamination from certain fish is low in Ireland [35], the promotion of fish consumption should also emphasise general guidelines and guidelines for at risk groups, including pregnant women and women of reproductive age.

The analysis of other potential sources of exposure revealed that levels among those reporting exposure were generally below the US EPA [23] value, and were not significantly higher than those that were not exposed to other sources. While non dietary exposure to mercury is likely to be of minor importance [5], it is important to ensure that exposure from other sources is minimised. The majority of mercury emissions in Ireland are produced from solid waste incineration and fossil fuel combustion, particularly from coal burning power plants [37]. Proximity to such facilities was not recorded in the current study. Mercury exposure for those living near such facilities would need to be monitored in the future to ensure that levels are within recommended guidance values. Despite the relatively low risk to health in Ireland from mercury exposure, if mercury levels were further reduced, significant social and economic benefits could be achieved. For example, based on the calculated current life-time income benefits of a higher IQ, data from the study has been used to estimate an economic benefit of €22–24 million to Ireland if current exposure levels were reduced [19]. The Minimata Convention on Mercury [38] (signed by Ireland and 90 countries) requires countries to reduce mercury emissions. Although those reporting exposure to other potential sources of mercury contamination all had mercury levels below guidance values, it is worth noting that the highest levels were for those exposed to chemical hair treatment (mothers), those with amalgam teeth fillings (mothers and children), and those that had a mercury thermometer broken at home (children). These levels were unrelated to fish consumption. Despite the fact that the numbers exposed to such sources were relatively small (with the exception of amalgam teeth fillings) it would be important that families are aware of the risks of exposure from these sources. Mercury is banned from hair products in Ireland [39]. Further monitoring and testing of hair products such as those for chemical hair structure and treatment may be required to ensure that they do not contain mercury. In terms of amalgam teeth fillings, the low level of exposure in our study would not warrant remedial action. There is a lack of evidence to support the removal of amalgam fillings. The removal process generates mercury vapour which can increase exposure levels [40].

It must be acknowledged that the study does have a number of limitations. The sample size of120 mother/child pairs is not statistically representative of Ireland overall, nor in terms of age or gender. This limits the extrapolation of the findings to other social groups. Although the study achieved its quota of mother/child pairs, only 30% of parents were interested in participating in the study. If human biomonitoring were to be introduced on a wider scale, the recruitment process would need to be examined to help ensure a statistically representative sample. The survey tool employed was limited in terms of the level of detail it elicited (e.g., no data on the number of amalgam fillings, proximity to fossil fuel power stations). Nevertheless the findings do provide a valuable insight into mercury exposure in Ireland. Future monitoring should aim to provide nationally representative data which would facilitate a more robust analysis.

## 4. Conclusions

The study has shown the value of human biomonitoring to identify and manage both population and individual exposure risk. It is clear that hair mercury levels among a sample of mothers and children in Ireland are below the health based guidance values of exposure. Although hair mercury levels were significantly higher in those that frequently consumed fish, these were also below guidance values. A system of regular human biomonitoring can help achieve reductions by measuring trends over time. Areas can then be targeted to help minimise their potential harmful effects on population health.
